# Computer-assisted navigation for removal of the foreign body in the lower jaw with a mandible reference frame: A case report

**DOI:** 10.1097/MD.0000000000018875

**Published:** 2020-01-17

**Authors:** Shuo Chen, Ying-Heng Liu, Xin Gao, Chan-Yuan Yang, Zhi Li

**Affiliations:** a Department of Oral and Maxillofacial Surgery; b The State Key Laboratory Breeding Base of Basic Science of Stomatology (Hubei-MOST) and the Key Laboratory of Oral Biomedicine Ministry of Education, School and Hospital of Stomatology, Wuhan University, Wuhan, People's Republic of China.

**Keywords:** foreign body, lower jaw, mandible, navigation system, reference frame

## Abstract

**Rationale:**

In surgery of the lower jaw, the application of computer-assisted navigation is complicated and challenging due to the mobile nature of the mandible. In this study, we presented a computer-assisted navigation surgery for removal of the foreign body in the lower jaw with a mandible reference frame, basing on the strategy that the mandible is independent as an entity.

**Patient concerns:**

A 41-year-old male patient, identified as having a broken fissure bur that displaced into the mandibular lingual soft tissue, was referred to our department. The fissure bur broke accidentally and then displaced into the soft tissue when the patient underwent extraction of the left mandibular impacted third molar.

**Diagnosis:**

A metallic foreign body in the left lower jaw, confirmed by orthopantomography.

**Interventions:**

A computer-assisted navigation surgery with a customized mandible reference frame.

**Outcomes:**

The broken bur was removed successfully. Satisfactory wound healing and mouth opening was achieved, without postoperative complications.

**Lessons:**

Surgeons should be alert to the presence of broken bur in the lower jaw and avoid its displacement into deep facial space, and computer-assisted navigation with a mandible reference frame is recommended for removal of the foreign body in the lower jaw.

## 1 Introduction

Originally implemented into neurosurgery in 1980s, computer-assisted navigation technology has subsequently been introduced into the field of oral and maxillofacial surgery.^[1–3]^ For the foreign body removal surgery in this field, this technology is helpful to identify the location of the foreign body and determine the optimal approach. The accuracy and effect of the operation can be greatly improved by this technology. This technology is widely reported on removal of foreign bodies in the maxillofacial region.^[3–12]^

Unlike other bones in the maxillofacial region, the mandible is an independent and movable body. In surgery of the lower jaw, the application of computer-assisted navigation is more complicated due to the mobile nature of the mandible.^[1]^ Previously, there were different solutions proposed by other researchers on navigation surgery in the lower jaw. Due to its specific independence among the maxillofacial bones, we think that the mandible can be regarded as an independent entity to perform navigation surgery. According to this strategy, the navigation reference frame is fixed directly on the surface of the mandible, allowing the navigation system to treat the mandible as an independent entity. In this case report, this new method was used to remove a broken fissure bur in the lower jaw. We conclude that this strategy successfully addressed the limitations of navigation surgery in the lower jaw.

## 2 Case report

A 41-year-old male patient, identified as having a broken fissure bur that displaced into the mandibular lingual soft tissue, was referred to our department. The fissure bur broke accidentally and displaced into the soft tissue when the patient underwent extraction of the left mandibular impacted third molar. The dentists in the local hospital attempted to retrieve the broken fissure bur, but they did not succeed. A mild contusion on the left side of the patient's mouth can be found, with a limited mouth opening (2 cm) and numbness on the left side of the lower lip. A wound after the tooth extraction was presented in the posterior area of the lower molars. The Ethics Committee of School and Hospital of Stomatology, Wuhan University gave approval for this case report. Written informed consent was obtained from the patient for publication of this case report and any accompanying images.

**Figure 1:**
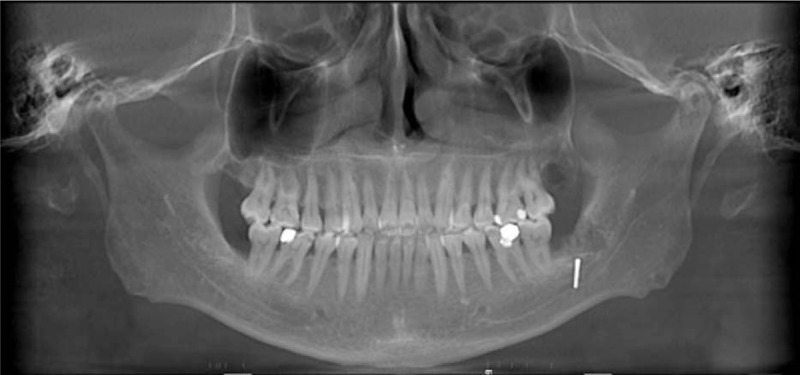
A metallic foreign body in the left lower jaw was presented in the orthopantomography.

An orthopantomography (OPG) confirmed the presence of a metallic foreign body in the left lower jaw (Fig. 1). In order to locate the foreign body accurately and avoid secondary injury, a computer assisted navigation surgery was scheduled for this patient.

**Figure 2:**
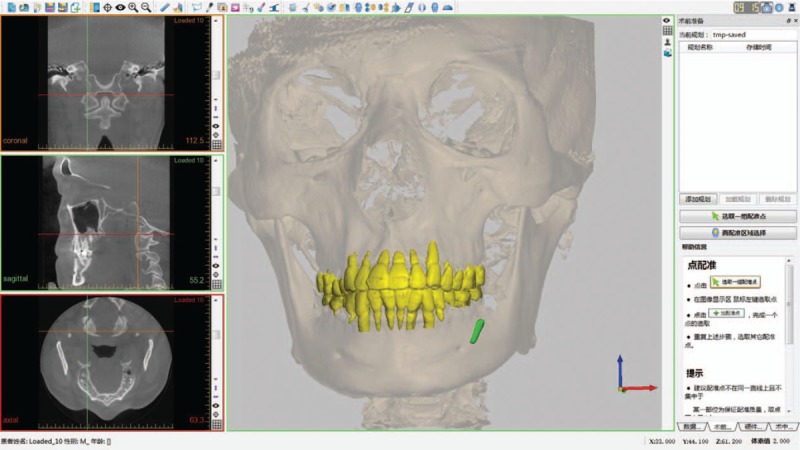
In the pre-surgical plan, the foreign body was labeled to generate the stereoscopic image.

The navigation system (AccuNavi-A, UEG Medical, China) was used in this case, and a customized mandible reference frame was prepared for the navigation surgery in the lower jaw. After the CT taken preoperatively, continuous fine-cut (0.625 mm) CT data were imported to the system to accomplish presurgical planning, in which the foreign body was labeled in the axial plane to generate the stereoscopic image of the broken bur (Fig. 2).

**Figure 3:**
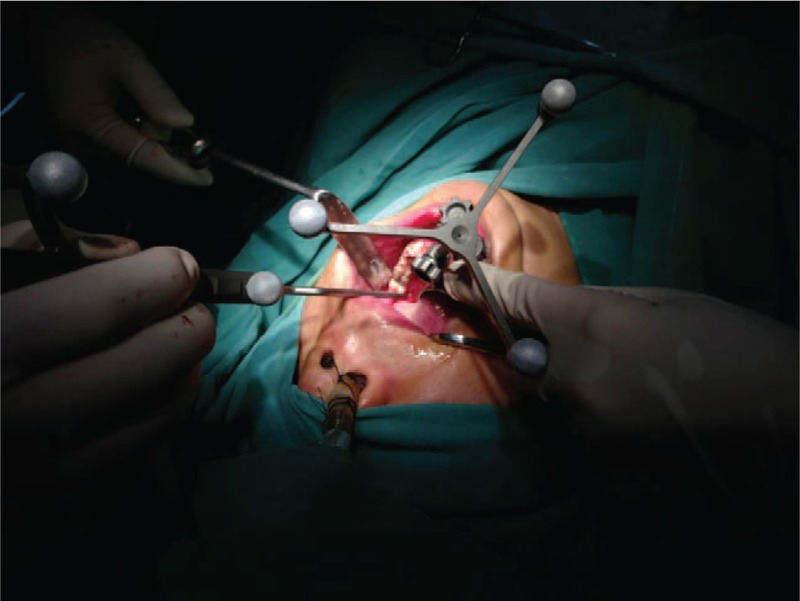
The customized reference frame was fixed rigidly to the patient's mandible, and the navigation surgery was performed while the mouth was open.

**Figure 4:**
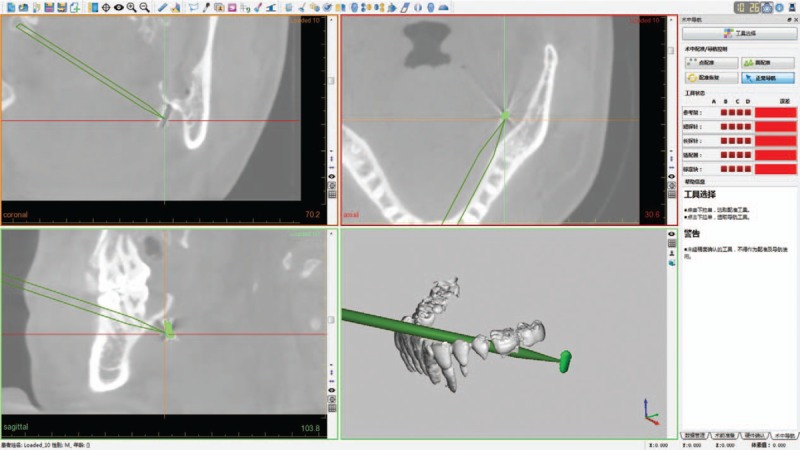
Real-time screenshot of the probe reaching the object under guidance of the navigation system.

**Figure 5:**
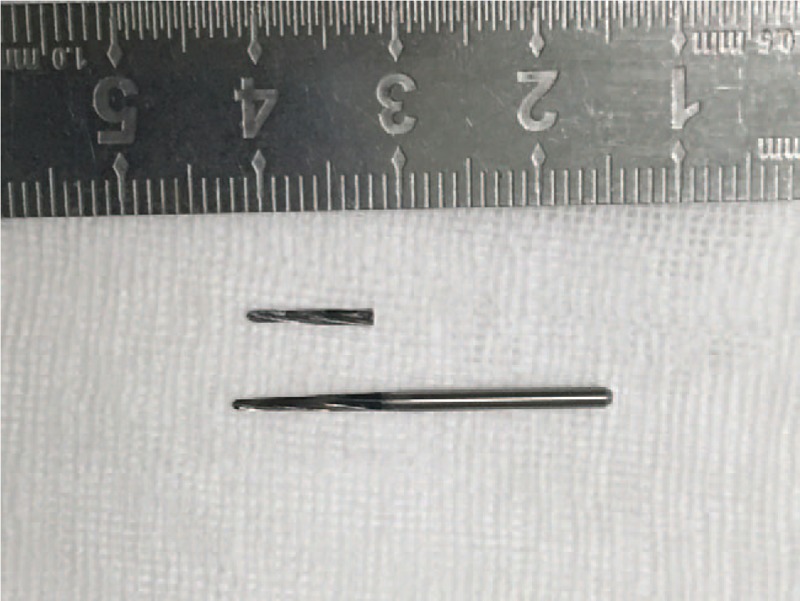
The foreign body taken out from this patient was confirmed to be the tip of a conventional high-speed fissure bur.

In the surgery, a vestibular incision (0.5 cm long) was made on the region of the right canine, exposing the mandible surface. By this approach, a customized reference frame was fixed rigidly to the patient's mandible (Fig. 3). The teeth in the mandible were selected by navigation probe, and the registration by point matching method was completed, whose accuracy (0.8 mm) was verified using the navigation pointer. The navigation surgery was performed while the mouth was open, and the suture at the original wound was first removed. Afterwards, the navigation probe was put in through the original wound to detect the foreign body (Fig. 4). The tip position and orientation of the probe was viewed continuously on screen of the navigation system. Under the guidance of the navigation system, the tip of the probe was inserted accurately, reaching the foreign body, which tilted in the lingual deep side of left mandibular extraction socket, with one end adjacent to the lingual bone plate and the other 4 mm away. The foreign body was finally clamped and removed successfully by the forceps. It was confirmed to be the tip of a conventional high-speed fissure bur (Fig. 5).

The broken bur was removed successfully, and there was no sign of lingual nerve palsy or infection when the patient was discharged. The time duration of the surgery using navigation system was 60 minutes. During the month-long follow-up visit, satisfactory wound healing and mouth opening was achieved, without postoperative complications.

## 3 Discussion

Some maxillofacial foreign body injuries are caused by iatrogenic factors, such as the displacement of the impacted teeth into the facial space and implants into the sinuses, etc. Broken dental bur displaced into the maxillofacial space during dental practice is a rare event, which is often stressful for the patient and dentist alike. Removal of maxillofacial foreign bodies generally follows the principles including early removal, hidden incision, minimal invasion, rapidity, and no damage to surrounding tissues.

Computer-aided navigation technology, a good method for detecting and localizing objects, has been widely applied in the removal of foreign bodies in maxillofacial region. However, this method has certain limitations for the removal of foreign bodies in some anatomical structures with mobile nature, such as the mandible and the soft tissue of the lower jaw. The cause is the topographic changes triggered by surgery, resulting in discrepancies between the preoperative image data and the surgical site. For navigation surgery, image data acquired preoperatively should always accurately represent the structure of the tissue during the operation. The mobile nature of the mandible complicates its synchronization with the preoperative image data during the navigation surgery. Currently, there are three possible solutions for navigation surgery in the lower jaw.

The first option is intermaxillary fixation. Lin et al reported a case of intraoperative navigation for mandibular angle surgery, in which they used a steel wire for intermaxillary fixation, to ensure that the upper and lower jaws were in an intercuspal position.^[13]^ Due to the unique reproducibility of the jaw, the uniform synchronization of the position is guaranteed in the process of preoperative CT scan and navigation in surgery. However, this method greatly restricts the access to the surgical site. Furthermore, it is not available for surgery via the intraoral approach.

The second approach is to use a specific occlusal splint to hold the upper and lower jaws on a fixed position repetitively. Günter et al designed a dental acrylic splint with an embedded screw and kept the patient wearing it during process of the CT scan as well as the operation.^[14]^ This splint was designed to fix the mandible in a reproducible position (maximal mouth opening). By this method, a mini screw left in the condyle was removed successfully via a minimally invasive approach. Li et al designed an open splint situated between the upper and lower dentition to fix the mandible at a specific open site, removing dental materials displaced into the mandible successfully by the mandibular navigation.^[4]^ On the surface, artificial fixation of the mandible with splints does not introduce additional errors, but this method increases the sensitivity of the relative movement of the mandible, which in turn reduces the accuracy of the navigation system.^[1,2,12]^

The third method is to install a dynamic reference frame in the mandible so that the jaw movement and position can be continuously tracked during the operation. Casap et al evaluated the accuracy and suitability of two computer navigation systems for mandibular surgery (Image-Guided Implantology system and LandmarX system).^[1]^ They concluded that direct tracking of the mandible via a tooth-mounted sensor frame and tooth-supported fiducial markers was useful for mandibular navigation and superior to indirect tracking in surgery of the lower jaw. However, fixation of tooth-mounted sensor frame and tooth-supported fiducial markers requires special instruments and the process is more time-consuming and complicated. In addition, these special instruments may affect the operative procedures to some extent and possibly lose its position, which could lead to failure of the navigation surgery.

In this case, in order to locate foreign bodies more accurately and avoid secondary injury resulted from blind exploration, we chose to use computer-assisted navigation technology via the original wound of tooth extraction. The removal of the foreign bodies via intraoral approach requires the patient to be in a mouth-opening state during the operation, and thus the method of intermaxillary fixation cannot be used. Basing on the result of this case, we believe that the mandible can be treated as a single entity, being independent from the whole skull, to perform the navigation surgery in the lower jaw. By this method, the navigation reference frame is directly fixed in the mandible to track it, as the foreign bodies remaining relatively stationary with the mandible in the adjacent soft tissues. In addition, the use of reference frame solved the problem of the requirement for special instruments or devices, making the surgical procedures more timesaving and easier.

In conclusion, the strategy that treating the mandible as an independent entity is an effective solution for computer-assisted navigation surgery to remove the foreign body in the lower jaw. It is reasonable to believe that this method is suitable for navigation surgeries in the lower jaw.

## Author contributions

**Conceptualization:** Zhi Li.

**Formal analysis:** Zhi Li.

**Investigation:** Chan-Yuan Yang, Zhi Li.

**Methodology:** Shuo Chen, Ying-Heng Liu, Chan-Yuan Yang, Zhi Li.

**Writing – original draft:** Shuo Chen, Ying-Heng Liu, Zhi Li.

**Writing – review & editing:** Zhi Li.

Zhi Li orcid: 0000-0003-1927-9294.

## References

[1] Casap N Wexler A Eliashar R Computerized navigation for surgery of the lower jaw: comparison of 2 navigation systems. J Oral Maxillofac Surg 2008;66:1467–75. 18571032 10.1016/j.joms.2006.06.272

[2] Yu H Shen SG Wang X The indication and application of computer-assisted navigation in oral and maxillofacial surgery Shanghai's experience based on 104 cases. J Craniomaxillofac Surg 2013;41:770–4. 23462802 10.1016/j.jcms.2013.01.016

[3] Heiland M Habermann CR Schmelzle R Indications and limitations of intraoperative navigation in maxillofacial surgery. J Oral Maxillofac Surg 2004;62:1059–63. 15346354 10.1016/j.joms.2004.02.013

[4] Li P Li Z Tian W A strategy for removal of foreign body in mandible with navigation system. Int J Oral Maxillofac Surg 2015;44:885–8. 25744644 10.1016/j.ijom.2015.01.021

[5] Siessegger M Mischkowski RA Schneider BT Image guided surgical navigation for removal of foreign bodies in the head and neck. J Craniomaxillofac Surg 2001;29:321–5. 11777348 10.1054/jcms.2001.0254

[6] Ewers R Schicho K Undt G Basic research and 12 years of clinical experience in computer-assisted navigation technology a review. Int J Oral Maxillofac Surg 2005;34:1–8. 15617960 10.1016/j.ijom.2004.03.018

[7] Eggers G Haag C Hassfeld S Image-guided removal of foreign bodies. Br J Oral Maxillofac Surg 2005;43:404–9. 15908086 10.1016/j.bjoms.2005.01.016

[8] Gui H Yang H Shen SG Image-guided surgical navigation for removal of foreign bodies in the deep maxillofacial region. J Oral Maxillofac Surg 2013;71:1563–71. 23810618 10.1016/j.joms.2013.04.001

[9] Lee TY Zaid WS Broken dental needle retrieval using a surgical navigation system: a case report and literature review. Oral Surg Oral Med Oral Pathol Oral Radiol 2015;119:e55–9. 25442246 10.1016/j.oooo.2014.08.019

[10] Yang CY Yang RT He SG Removal of a large number of foreign bodies in the maxillofacial region with navigation system. Dent Traumatol 2017;3:230–4. 10.1111/edt.12313 27926993

[11] Campbell A Costello BJ Retrieval of a displaced third molar using navigation and active image guidance. J Oral Maxillofac Surg 2010;68:480–5. 20116728 10.1016/j.joms.2009.06.032

[12] Kang SH Kim MK Kim JH The validity of marker registration for an optimal integration method in mandibular navigation surgery. J Oral Maxillofac Surg 2013;71:366–75. 22695020 10.1016/j.joms.2012.03.037

[13] Yanping L Xiaojun C Ming Y A pilot application of image-guided navigation system in mandibular angle reduction surgery. J Plast Reconstr Aesthet Surg 2010;63:e593–6. 20031490 10.1016/j.bjps.2009.11.043

[14] Schultes G Zimmermann V Feichtinger M Removal of osteosynthesis material by minimally invasive surgery based on 3-dimensionalcomputed tomography-guided navigation. J Oral Maxillofac Surg 2003;61:401–5. 12618985 10.1053/joms.2003.50067

